# Fertilizer application parameters for drip-irrigated peanut based on the fertilizer effect function established from a “3414” field trial

**DOI:** 10.1515/biol-2022-0894

**Published:** 2024-07-16

**Authors:** Zhijian Gao, Xinlu Bai, Xiaoyun Tang, Jinhu Zhi, Yu Liu, Guodong Wang, Guojiang Yang, Yantao Liu, Liang Wang

**Affiliations:** Xinjiang Academy of Agricultural and Reclamation Science, Shihezi, 832000, Xinjiang, China; Key Laboratory of Northwest Oasis Water-Saving Agriculture, Ministry of Agriculture and Rural Affairs, Shihezi, 832000, Xinjiang, China; Key Laboratory of Efficient Utilization of Water and Fertilizer, Xinjiang Production & Construction Corps, Shihezi, 832000, Xinjiang, China; Agricultural College of Tarim University, Alar, 843300, China; Shihezi Academy of Agriculture Science, Shihezi, 832000, Xinjiang, China

**Keywords:** peanut yield, mulched drip irrigation, “3414” field trial, fertilizer effect model, recommended fertilization

## Abstract

Scientific fertilization is an important technical means of achieving high and stable peanut yields. Using soil testing and formula fertilization, the “3414” optimal regression design was used and included 14 nitrogen (N), phosphorus (P), and potassium (K) fertilization treatments. Ternary quadratic functions of the fertilizer effect were established according to three-season field experiments and the regression analysis of fertilizer–yield function was performed to explore the optimal fertilizer application mode and ratio for peanuts under mulched drip irrigation (MDI), and a suitable fertilizer application system was established. The ternary quadratic equation relating peanut yield (*y*) and the fertilizer application rates of N (N), P (P_2_O_5_), and K (K_2_O) was obtained after fitting, i.e., *y* = 2912.528 + 21.432N + 16.324P + 6.181K − 0.051N^2^ − 0.109P^2^ − 0.061K^2^ + 0.017NP + 0.023NK + 0.086PK, and significance analysis and typicality assessment were performed. The model *R* ^2^ was 0.9709, both values are extremely significant (*p* < 0.01), which indicates that the obtained ternary quadratic fertilizer effect function is typical and could be used for statistical purposes and fertilization recommendations. Three quadratic fertilizer effect functions were obtained. Among them, the equation for *K* is extremely significant, and the equations of *N* and *P* are significant. According to the assumption that the marginal yield is zero and the marginal profit is zero, the fertilizer application rate with the maximum yield, the fertilizer application rate with the best economic benefits, and the corresponding yields were obtained. The optimal fertilizer application rate predicted by the ternary quadratic fertilizer effect function was relatively high, so the three quadratic fertilizer effect functions were used for prediction. Under the test conditions, the recommended fertilizer application rates for peanuts under MDI are 256.6 kg N per ha, 164.2 kg P_2_O_5_ per ha, and 213.2 kg K_2_O per ha, the recommended fertilization ratio is 1:0.64:0.83, and the recommended ratio under formula fertilization is 23:15:19. The study has developed a data-based decision support system for Xinjiang drip-irrigated peanut, which assists farmers and agricultural managers in making more scientific and precise fertilization decisions based on the specific growth requirements of the crops and soil conditions. This evidence-based methodology enhances the precision of agricultural management, which is conducive to increasing crop yields while reducing resource wastage and environmental impact. However, multipoint and multiyear experiments are still needed to ensure that the findings are adaptable to the diverse soil conditions and fluctuating climate patterns that may be encountered in practice.

## 1 Introduction

In soil testing and formula fertilization, a ternary quadratic polynomial function of fertilizer effects is often used to establish nitrogen (N), phosphorus (P), and potassium (K) fertilizer effect equations and calculate recommended fertilizer application rates [1]. Many scholars have used “3414” field trials to establish fertilization index systems for wheat [2], corn [3], rice [4], and cotton [5] to determine recommended fertilization schemes. Qi et al. [6], Zhong et al. [7], and Li et al. [8] carried out fertilizer effect model studies on peanut, they recommended similar fertilization ratios for N and K but came to different conclusions regarding P fertilizer application, which may be due to regional variations. Some scholars have proposed fertilization based on the principle of combined organic and inorganic fertilization and the balanced application of macro and micro elements [9] or on reducing N and P fertilizer application and focusing on K fertilizer [10]. Others have explored the theory and application of whole-process controlled fertilization, i.e., “N application in the initial stage, calcium (Ca) application in the middle stage, and N application in the later stage”, to elucidate the governing principles of peanut fertilizer application [11]. Regardless of which fertilization technologies and principles are adopted, the basic soil fertility parameters and fertilization effect of the local area should be clarified.

Xinjiang has a long history of peanut planting. Peanut has been regarded as a secondary cash crop, and little research has been carried out, moreover, relevant technical parameters and models for water and fertilizer application are lacking. In recent years, under the background of the restructuring of the agricultural industry, rapid development of animal husbandry, and emergence of the economic and ecological benefits of peanut planting in the Xinjiang Production and Construction Corps (XPCC), the peanut planting area in the XPCC has gradually increased, the income of farmers in Xinjiang has gradually increased as a result of peanut cultivation, and peanut has become an important cash crop with ecological benefits. The water resources in Xinjiang are limited, and drip irrigation technology, as an efficient water-saving irrigation method, can effectively improve the utilization efficiency of water resources and promote increased agricultural production [12]. However, there are few reports on the “3414” field trials of drip irrigated peanut cultivation in Xinjiang, where most growers rely on experience for fertilization. Therefore, excessive application of fertilizer often occurred, leading to significant nutrient loss and environmental pollution.

In this study, based on the unique climatic conditions of Xinjiang and conducted “3414” field trials of peanut planting, the technical parameters related to water and fertilizer application for drip-irrigated peanuts under mulched drip irrigation were explored to establish and improve a high-quality, high-yield, and high-efficiency cultivation model for drip-irrigated peanut. The aim of this study was to clarify the optimal fertilizer application rate and fertilization parameters for local peanut planting through experiments to improve traditional water and fertilizer management methods and to continuously improve resource use efficiency and reduce environmental pollution while increasing production. This study is expected to provide a theoretical basis for the high-yield cultivation of drip-irrigated peanuts in Xinjiang and provide technical support for modifying structural components of agriculture, developing animal husbandry, increasing employees’ income and increasing field efficiency in the XPCC.

## 2 Materials and methods

### 2.1 Overview of the test sites

The experiments were conducted at the Shihezi Scientific Observatory Experimental Station for Efficient Water Use in Crops of the Ministry of Agriculture and Rural Affairs (Shihezi, Xinjiang, 45°38′N, 86°09′E). The area has a typical arid and semiarid continental climate, with scarce precipitation, dry air, and concentrated light and heat. The annual average temperature is 6.5–7.2℃, the annual average precipitation is 115 mm, the evaporation is 1,942 mm, and the annual sunshine duration is 2,526–2,874 h. The sunshine duration in growing season is 1,900–2,000 h, the annual frost-free period is approximately 160 days, and the accumulated temperature ≥10℃ is 3,570–3,729℃. The experimental field soil is irrigated grey desert soil with uniform soil fertility. The soil physical and chemical characteristics at a depth of 0–20 cm were as follows: organic matter 10.58 g C kg^−1^, alkali-hydrolysable N 41.55 mg N kg^−1^, available P 18.38 mg P kg^−1^, available K 201.5 mg K kg^−1^, soil pH 8.4, soil bulk density 1.17 g cm^−3^, and field water holding capacity 21.03%. The experiments were conducted during 2016–2018, and sowing was performed on approximately May 10 every year. The peanut variety was Huayu 33 (Shandong Peanut Research Institute), and the test fertilizers were urea CO(NH_2_)_2_ (46–0–0), calcium superphosphate Ca(H_2_PO_4_)_2_ (0–12–0), and potassium sulfate K_2_SO_4_ (0–0–50). Mulch with a width of 145 cm was used, and hole sowing was conducted with four planting rows per mulch strip. The row spacing within the mulch strip was 35 cm + 45 cm + 35 cm, the spacing between plants was 15 cm, the row spacing between adjacent rows was 70 cm, and the theoretical density was 147,000–156,000 holes ha^−1^. Single-wing labyrinth drip irrigation tape was used with a drip tape emitter spacing of 30 cm and a flow rate of 2.0 L h^−1^. The agronomic measures were consistent with those in field management mode.

### 2.2 Experimental design

The 3414 experiment is a design method for field trials on the effects of fertilizers, involving three factors: N, P, and K, with each factor having four different application rates, resulting in 14 treatment combinations. This design allows researchers to assess the impact of different fertilization rates and ratios on crop yield, as well as to establish a fertilizer effect equation, which is crucial for understanding the influence of fertilization on crop yield. The 14 treatments are: (1) N_0_P_0_K_0_, (2) N_0_P_2_K_2_, (3) N_1_P_2_K_2_, (4) N_2_P_0_K_2_, (5) N_2_P_1_K_2_, (6) N_2_P_2_K_2_, (7) N_2_P_3_K_2_, (8) N_2_P_2_K_0_, (9) N_2_P_2_K_1_, (10) N_2_P_2_K_3_, (11) N_3_P_2_K_2_, (12) N_1_P_1_K_2_, (13) N_1_P_2_K_1_, and (14) N_2_P_1_K_1_ (the subscripts indicate the level of fertilizer application, level 0 refers to no fertilization, level 2 refers to the optimal local fertilizer application rate, level 1 = level 2 × 0.5, level 3 = level 2 × 1.5) (Table 1). 20% N fertilizer, 50% P fertilizer, and K fertilizer are applied as base fertilizer. The remaining fertilizer as top dressing divided into four equal rates for application was applied by a Venturi injector connected to the drip irrigation system. The experiments were repeated three times and arranged in random blocks, and the soil conditions within the blocks were relatively consistent. The area of the plot was 5 m × 10 m = 50 m^2^, and protection rows were set up outside the test area. To determine harvest yield, the fresh weight and dry weight of pods in each plot were weighed, and the yield per unit area was calculated. All fertilizers were applied with water droplets. Table 2 shows the distribution of growth periods.

**Table 1:** Experimental design and fertilizer application rates

Treatment	Fertilizer application rate (kg ha^−1^)		
	N	P_2_O_5_	K_2_O
N_0_P_0_K_0_	0	0	0
N_0_P_2_K_2_	0	180	225
N_1_P_2_K_2_	135	180	225
N_2_P_0_K_2_	270	0	225
N_2_P_1_K_2_	270	90	225
N_2_P_2_K_2_	270	180	225
N_2_P_3_K_2_	270	270	225
N_2_P_2_K_0_	270	180	0
N_2_P_2_K_1_	270	180	112.5
N_2_P_2_K_3_	270	180	337.5
N_3_P_2_K_2_	405	180	225
N_1_P_1_K_2_	135	90	225
N_1_P_2_K_1_	135	180	112.5
N_2_P_1_K_1_	270	90	112.5

**Table 2:** Fertilizer distribution ratios of drip-irrigated peanuts at different growth stages

Growth stage	Number of applications (times)	Irrigation quota (m^3^ ha^−1^)	Fertilizer ratio (%)		
			N	P_2_O_5_	K_2_O
Basal fertilizer	—	—	—	50	—
Seedling stage	1	150–225	5	—	—
Flowering-pegging stage	4	375–450	45	45	45
Pod bearing stage	3	300–375	35	50	50

### 2.3 Calculation method

#### 2.3.1 Typicality determination

According to the unconstrained optimization method, the principal minors of each order of the Hessian matrix *G*(*X*′) are set to *G*_1_, *G*_2_, and *G*_3_, and then the values of the determinants are

\[{G}_{1}=\text{}2{b}_{4},]\]

\[{G}_{2}=\text{}4{b}_{4}{b}_{5}-b{7}^{2},]\]

\[{G}_{3}=\text{}2(4{b}_{4}{b}_{5}{b}_{6}+\text{}{b}_{7}{b}_{8}{b}_{9}-{b}_{4}{{b}_{9}}^{2}-{b}_{5}{{b}_{8}}^{2}-{b}_{6}{{b}_{7}}^{2}).]\]

If *g*(*x*′) = 0, *G*_1_ < 0, *G*_2_ > 0, and *G*_3_ < 0, then matrix *G*(*X*′) is negative definite, and the fertilizer effect model has a maximum value. Furthermore, if the values of N, P, and K corresponding to point *x*′ represented by *g*(*x*′) = 0 are all within the range of the designed fertilizer application rates in this study, then the model is typical [13].

#### 2.3.2 Recommended fertilizer application rate

The peanut yield was set as the dependent variable *y*, and the application rate of each fertilizer was set as the independent variable *x*, and then ternary quadratic and quadratic fertilizer effect functions were obtained after fitting. After comprehensive consideration of typicality, the *F* test, and the *R* ^2^ test, the maximum fertilizer application rate and the fertilizer application rate with best economic benefit, as well as the corresponding yields, were calculated to determine the recommended fertilizer application rate for peanuts.

### 2.4 Statistical analysis

The test data were analyzed using Excel 2007, and the graphs were plotted using SigmaPlot 12.5.

## 3 Results

### 3.1 Relationships between fertilizer application rates and peanuts yield

#### 3.1.1 Analysis of the results of five conventional treatments

Treatments 1, 2, 4, 6, and 8, i.e., the five conventional treatments in the “3414” field trial, corresponded to no fertilizer, N deficiency, P deficiency, full fertilizer, and K deficiency, respectively. The level “2” fertilizer application rate was used in each treatment. For the five conventional treatments, the effects of N, P, and K fertilizers on increasing yield were investigated in the field trials (Table 3). Table 4 shows that the yield increase and yield increase rate of treatment 6 (full fertilizer) and treatment 1 (no fertilizer) were the highest at 5844.0 kg ha^−1^ and 194.31%, respectively, and the yield increase rates of the N deficiency, P deficiency, and K deficiency treatments were 47.12, 52.36, and 59.37%, respectively, indicating that under the experimental conditions, the dependence of drip-irrigated peanuts on N was higher than that on P and K.

**Table 3:** Comparison of five conventional treatments

Treatment	Yield (kg ha^−1^)	Increase in yield (kg ha^−1^)		Increase ratio (%)	
		No fertilizer	Full fertilizer	No fertilizer	Full fertilizer
N_0_P_0_K_0_	3007.5	—	—	—	—
N_0_P_2_K_2_	4170.5	1163.0	4681.0	38.67	47.12
N_2_P_0_K_2_	4635.0	1627.5	4216.5	54.11	52.36
N_2_P_2_K_2_	8851.5	5844.0	—	194.31	—
N_2_P_2_K_0_	5255.5	2248.0	3596.0	74.75	59.37

**Table 4:** Prediction results of the ternary quadratic effect equation

Forecast	Fertilizer application rate (kg ha^−1^)			Yield (kg ha^−1^)
	N	P_2_O_5_	K_2_O	
Maximum application rate and yield	295.0	194.8	245.0	8420.4
Optimal application rate and yield	287.2	190.3	255.2	8371.6

Note: The calculation assumed that the economic value of N fertilizer was 4 yuan kg^−1^, that of P fertilizer was 7.4 yuan kg^−1^, that of K fertilizer was 6.8 yuan kg^−1^, and that of the peanut product was 5.2 yuan kg^−1^.

#### 3.1.2 Fitting of the ternary quadratic effect function and prediction analysis

Regression analysis was performed using the ternary quadratic regression model *y* = *b*_0_ + *b*_1_N + *b*_2_P + *b*_3_K + *b*_4_N^2^ + *b*_5_P^2^ + *b*_6_K^2^ + *b*_7_NP + *b*_8_NK + *b*_9_PK in Microsoft Excel 2007. The following ternary quadratic effect function between peanut yield (*y*) and the application rates of N (N), P (P_2_O_5_), and K (K_2_O) fertilizer was obtained after fitting:

(1) \[y=2912.528\text{}+21.432\text{N}+16.324\text{P}+6.181\text{K}-0.051{\text{N}}^{2}-0.109{\text{P}}^{2}-0.061{\text{K}}^{2}+\text{}0.017\text{NP}+\text{}0.023\text{NK}+0.086\text{PK,}]\]

where *y* is the economic yield of peanut (kg ha^−1^) and N, P, and K are the application rates of N, P_2_O_5_, and K_2_O, respectively.

When N, P, and K were at the 0 level, the model constant term was 2912.528, which was basically consistent with the actual situation (3007.5 kg). Significance analysis of the effect equation was further performed. The results showed that the correlation of the fitted ternary quadratic effect function is extremely significant, *R* ^2^ = 0.9709, and the *F* test (*F* = 14.8722) showed that the regression relationship is extremely significant, indicating a good fit. According to the typicality determination, *G*_1_ = −0.204, *G*_2_ = 0.022, and *G*_3_ = −0.002 were obtained, and *G*(*X*′) is negative definite, *X*′ = (N, P, K) = (295.0, 194.8, 245.0), and the extreme points fell within the experimental design range. Therefore, equation (1) is a typical formula.

Assuming that the marginal product is zero and the marginal profit is zero, the fertilizer application rate corresponding to the maximum yield, the fertilizer application rate corresponding to the best economic benefits, and the corresponding yields were calculated (Table 4).

#### 3.1.3 Principal factor analysis

Since factors have different effects on yield, principal factor analysis must be performed to obtain the marginal effect of changes in each factor, and the value can directly reflect the impact of a certain experimental factor on the target of the function [14,15]. The dimensionality reduction method was used for the comprehensive mathematical model of yield, and the other factors were fixed at the zero level. The effect equations of single factors were as follows:

(2) \[{y}_{1}=\text{}2912.528+21.432\text{N}-0.051{\text{N}}^{2},]\]

(3) \[{y}_{2}=\text{}2912.528\text{}+\text{}16.324\text{P}-0.109{\text{P}}^{2},]\]

(4) \[{y}_{3}=\text{}2912.528\text{}+\text{}6.181\text{K}-0.061{\text{K}}^{2}.]\]

The coded values of the factors corresponding to the above formulas were substituted, and the results are shown in Table 5. The arithmetic mean marginal effect value of each factor shows that the order of the impact of each factor on yield is N > P > K; therefore, the key to increasing peanut yield in this area lies in the application rate of N fertilizer.

**Table 5:** Marginal effect value of each factor (N, P, and K)

Variable	Level 0	Level 1	Level 2	Level 3	Average
N	21.432	21.33	21.228	21.126	21.279
P	16.324	16.106	15.888	15.67	15.997
K	6.181	6.059	5.937	5.815	5.998

#### 3.1.4 Fitting of quadratic effect function and predictive analysis

**Figure 1:**
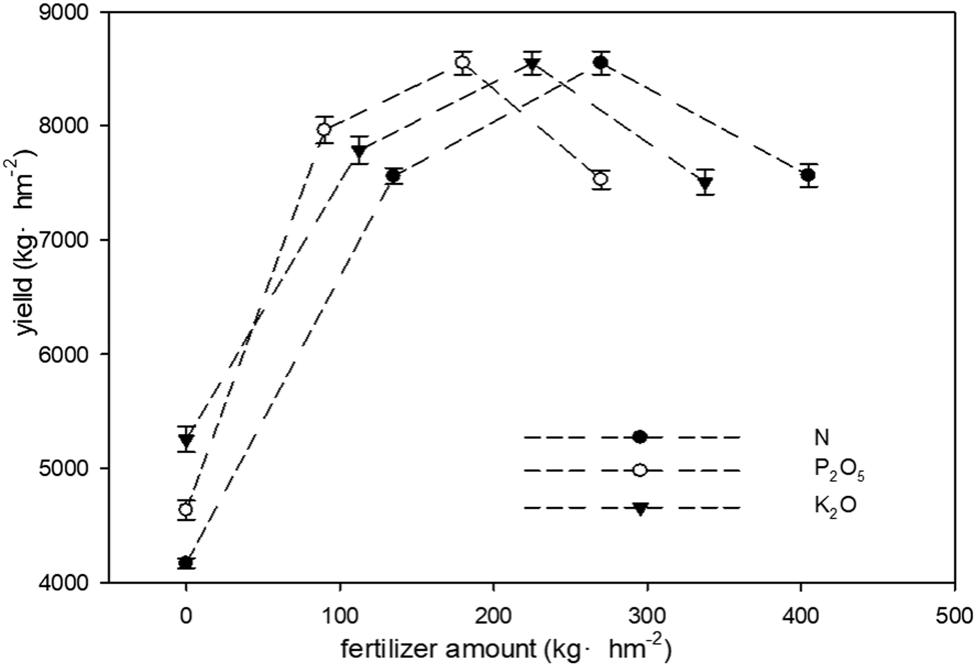
Univariate effect curves under different fertilization levels.

The quadratic fertilizer effect function was fitted to supplement and optimize the ternary quadratic fertilizer function [16]. The application rates and yields of each factor (fertilizer type) were selected. The data of treatments 2, 3, 6, and 11 were used for N, the data of treatments 4, 5, 6, and 7 were used for P, and the data of treatments 8, 9, 6, and 10 were used for K. The formula y = *a* + *bx* + *cx* ^2^ was used for the quadratic regression model, and the curves resulting from regression analysis were drawn. Each factor was successfully fitted by the quadratic effect equation, and the coefficients of the quadratic terms were less than zero, indicating that the parabola opens downward. The regression results are shown in Table 6 and Figure 1.

**Table 6:** Fitting results of the quadratic effect function

Factor	Quadratic effect equation	*R* square	*P* value
N	\[y\text{}=\text{}4191.375+32.5824x-0.0600{x}^{2}]\] (5)	0.9992	0.0138*
P	\[y=4691.500+46.5472x-0.1342{x}^{2}]\] (6)	0.9929	0.0334*
K	\[y=5253.700+30.5351x-0.0707{x}^{2}]\] (7)	0.9999	0.0009**

Note: The numbers within parentheses are the equation numbers. * indicates significance at the 0.05 level (*p* < 0.05), and ** indicates significance at the 0.01 level (*p* < 0.01).

Based on the quadratic effect equations, the partial derivative function was obtained according to the maximum marginal effect. The fertilizer application rate corresponding to the maximum yield was determined by the equation *b* + 2*ax* = 0, and then the maximum yield was predicted. The fertilizer application rate corresponding to the optimal economic benefit was obtained according to the equation *b* + 2*ax* = *Px*/*Py*, and then the optimal yield was predicted (Table 7).

**Table 7:** Prediction results for each factor obtained using the quadratic effect function (unit: kg ha^−1^)

Factor	Prediction	Fertilizer application rate (kg ha^−1^)			Yield (kg ha^−1^)
		N	P_2_O_5_	K_2_O	
N	Maximum application rate and yield	261.6	—	—	8613.7
	Optimal application rate and yield	256.6	—	—	8611.3
P	Maximum application rate and yield	—	170.6	—	8508.8
	Optimal application rate and yield	—	164.2	—	8503.2
K	Maximum application rate and yield	—	—	224.2	8780.2
	Optimal application rate and yield	—	—	213.3	8770.5

The equation shows that when N is at the 0 level, the constant term is 4191.375 (yield under N deficiency, equation (5)), when P is at the 0 level, the constant term is 4691.5 (yield under P deficiency, equation (6)), and when K is at the 0 level, the constant term is 5253.7 (yield under K deficiency, equation (7)). The model results and the actual situation are basically consistent: yield under K deficiency > yield under P deficiency > yield under N deficiency. The primary term coefficients reflect the yield-increasing effects of N, P, and K, and the values are 32.5824, 46.5472, and 30.5351, respectively, indicating that the yield-increasing effect is P fertilizer > N fertilizer > K fertilizer.

### 3.2 Research and development of a formula for fertilization for drip irrigation

Based on the quadratic effect equation, the partial derivative function was obtained according to the maximum marginal effect, and the optimal fertilizer application rates for drip-irrigated peanuts were obtained: 256.6 kg N ha^−1^ equation (5), (164).2 kg P_2_O_5_ ha^−1^ equation (6), and 213.3 kg K_2_O ha^−1^ equation (7), with a fertilization ratio of 1:0.64:0.83. The fertilizer formula was calculated using urea, monoammonium phosphate, and potassium chloride as raw materials.

#### 3.2.1 Effects of formula fertilization on the yield of drip-irrigated peanuts

As shown in Table 8, the number of pods in a single hole under formula fertilization increased by 4.4% compared to conventional fertilization treatment. The 100-pod weight with formula fertilization significantly increased by 2.97 g than that with conventional fertilization. Compared with conventional fertilization, the yield under formula fertilization significantly increased by 503.63 kg ha^−1^, or 6.3%.

**Table 8:** Effects of two kinds of fertilization on the yield and yield elements of drip-irrigated peanuts

Fertilization category	Number of holes (holes ha^−1^)	Number of pods in a single hole (count)	100-pod fresh weight (g)	100-pod dry weight (g)	Yield (kg ha^−1^)
Conventional fertilization	14.25 × 10^4^a	33.8a	165.7b	78.2a	7981.9b
Formula fertilization	14.25 × 10^4^a	35.3a	168.7a	77.9a	8485.5a

Note: Different letters in the same column indicate significant differences at the 5% level.

#### 3.2.2 Effects of formula fertilization on the fertilizer use efficiency of drip-irrigated peanuts

Fertilizer partial factor productivity (FPFP) is an important indicator to measure fertilizer efficiency. Table 9 shows that, compared with conventional fertilization, formula fertilization can save 5.0% of N, 8.7% of P, and 5.2% of K on average, the FPFP values of N, P, and K fertilizers under conventional fertilization were 29.56, 44.34, and 35.48 kg kg^−1^, respectively, while those of formula fertilization were 33.07, 51.67, and 39.78 kg kg^−1^, the FPFP values of formula fertilization were 11.9, 16.5, and 12.1% higher than those of conventional fertilization.

**Table 9:** Effects of two modes of fertilization on the fertilizer PFP of drip-irrigated peanuts

Fertilization type	Fertilizer application rate (kg ha^−1^)			PFP		
	N	P_2_O_5_	K_2_O	N	P_2_O_5_	K_2_O
Conventional fertilization	270.0	180.0	225.0	29.56a	44.34a	35.48a
Formula fertilization	256.6	164.2	213.3	33.07a	51.67a	39.78a

Note: Different letters in the same column indicate significant differences at the 5% level.

## 4 Discussion

Using the fertilizer effect function to establish fertilization recommendations is a classic method [17]. Previous studies have largely focused on the relationship between individual nutrients and yield, and the classic models of the relationship between nutrients and yield were established such as the parabolic models between nitrogen fertilizer and yield, and the models combining linear and plateau relationships between N fertilizer and yield [18]. The 3414 experiment provides us with a good method for simultaneously and comprehensively studying the efficacy of N, P, and K fertilizers. The results of fertilizer field trials are used to establish a biostatistical regression model, and the recommended fertilizer application rates for representative plots and fertilization parameters such as the interaction effect between fertilizers are calculated according to the model, this approach is intuitive, easy to implement, and conducive to macroscopic regulation [19]. Whether a fitted ternary quadratic effect function can be used to predict the fertilizer application rate and yield should be determined. Some researchers believe that, if the obtained ternary quadratic effect function follows a diminishing rate of return in fertilization, i.e., the equation coefficients b1, b2, and b3 are all positive, b4, b5, and b6 are all negative, and the F-test value is above the level of significance, then the equation is the typical fertilizer effect function [20,21,22,23], which can be used to recommend fertilizer application rates for agricultural production. This determination method has certain limitations, and an atypical model can be misrepresented as the typical model by extrapolating the maximum values. Although this type of model can provide the highest yield, extreme value points are outside the designed range of the fertilizer trial [24]; therefore, the conclusions are extrapolated and cannot be used in practice. This determination method can be used for preliminary determination, after removing atypical equations, the unconstrained optimization method can be used for accurate determination.

In this study, the maximum yield, the yield with the best economic benefits, and the corresponding N, P, and K fertilizer application rates predicted by the ternary quadratic function were all higher than those predicted by the quadratic function, which was consistent with previous studies reporting that the optimal fertilizer application rates calculated by successfully fitted ternary quadratic functions are always high [16,25]. Analysis of the principal factor effects of the ternary quadratic function and the quadratic effect function showed that the yield-increasing effect of P fertilizer > the yield-increasing effect of N fertilizer > the yield-increasing effect of K fertilizer and highlighted the importance of N fertilizer in obtaining a high yield of peanut. Overall, these results indicated that the key to improving peanut yield lies in N and P inputs and a rational application ratio of N, P, and K.

Relative yield, also known as the dependence rate of crops on soil fertility, is the ratio of the yield of a no-fertilizer area or fertilizer-deficient area to that of the full-fertilizer area. This value can reflect the degree to which basic soil fertility satisfies the nutrient demand of crops, act as a grading index of soil basal fertility [26], and be used to evaluate crop responses to fertilization [27]. From the perspective of relative yield and the criteria of nutrient abundance and deficiency indicators [28], a lack of N fertilizer directly affected peanut yield. Based on the optimal fertilizer application rate for peanuts obtained from the empirical ternary quadratic effect function, the corresponding fertilization ratio was 1:0.64:0.83. This method is affected by conditions such as soil, climate, and cultivation, has obvious regional characteristics, and lacks wide-scale consistency and repeatability [29]. Even the recommended values obtained with a given crop yield equation with the same fertilizer application rate vary due to changes in fertilizer and crop prices. The empirical fertilizer effect function is obtained by directly studying crops, so its metrological accuracy and authenticity are unmatched by other methods [1], and it is the main technical method used to achieve controlled fertilization in China and abroad [30]. The calculated ratio of the formula fertilization scheme using urea, monoammonium phosphate, and potassium chloride as raw materials was 23:15:19. Although the recommended fertilization rates and formula fertilization ratios have been obtained, further research may be needed to fine-tune these ratios to accommodate different soil conditions and climate changes. Multipoint and multiyear experiments are still needed to obtain accurate fertilization parameters. In addition, some new agricultural technologies, such as microbial inoculants and new non-destructive monitoring techniques, can be applied for the production of drip-irrigated peanuts in Xinjiang to ensure high quality and yield [31,32,33].

## 5 Conclusion

In the unique climate conditions of Xinjiang, a 3-year field experiment of “3414” drip irrigation peanut planting was conducted. The results showed that the fertilization ratio of drip-irrigated peanuts in Xinjiang was 1:0.64:0.83, the recommended fertilizer application rates were 256.6–295 kg N ha^−1^, 164.2–194.8 kg P_2_O_5_ ha^−1^, and 213.2–255.2 kg K_2_O ha^−1^, and the ratio of formula fertilization was 23:15:19. The ternary quadratic functions of the fertilizer effect established according to the fertilizer–yield function had a high fitting quality (*R* ^2^ = 0.9709). Meanwhile, multipoint and multiyear experiments are needed to obtain accurate fertilization parameters.
